# Supplementary material for: Adaptation to hydrostatic pressure modulates proteome dynamics in corrosive sulfate-reducing bacteria
Source: Microbiol Spectr. 2025 Nov 12;13(12):e01590-25. doi: 10.1128/spectrum.01590-25 (PMC12671193; doi:10.1128/spectrum.01590-25)
Supplement: Supplemental figure and tables — Figure S1; Tables S1 to S4. [file spectrum.01590-25-s0001.docx]

**ADAPTATION TO HYDROSTATIC PRESSURE MODULATES PROTEOME DYNAMICS IN CORROSIVE SULFATE-REDUCING BACTERIA**

Nicolò Ivanovich^1^, Xue Guo^2^, Radoslaw M. Sobota^2^, Federico M. Lauro^1,^ ^3^

^1^Singapore Centre for Environmental Life Sciences Engineering, Nanyang Technological University, Singapore, Singapore

^2^Institute of Molecular and Cell Biology (IMCB), Agency for Science, Technology and Research (A*STAR), Singapore, Singapore

^3^Luminis Water Technologies, Singapore, Singapore

**SUPPLEMENTAL MATERIAL**


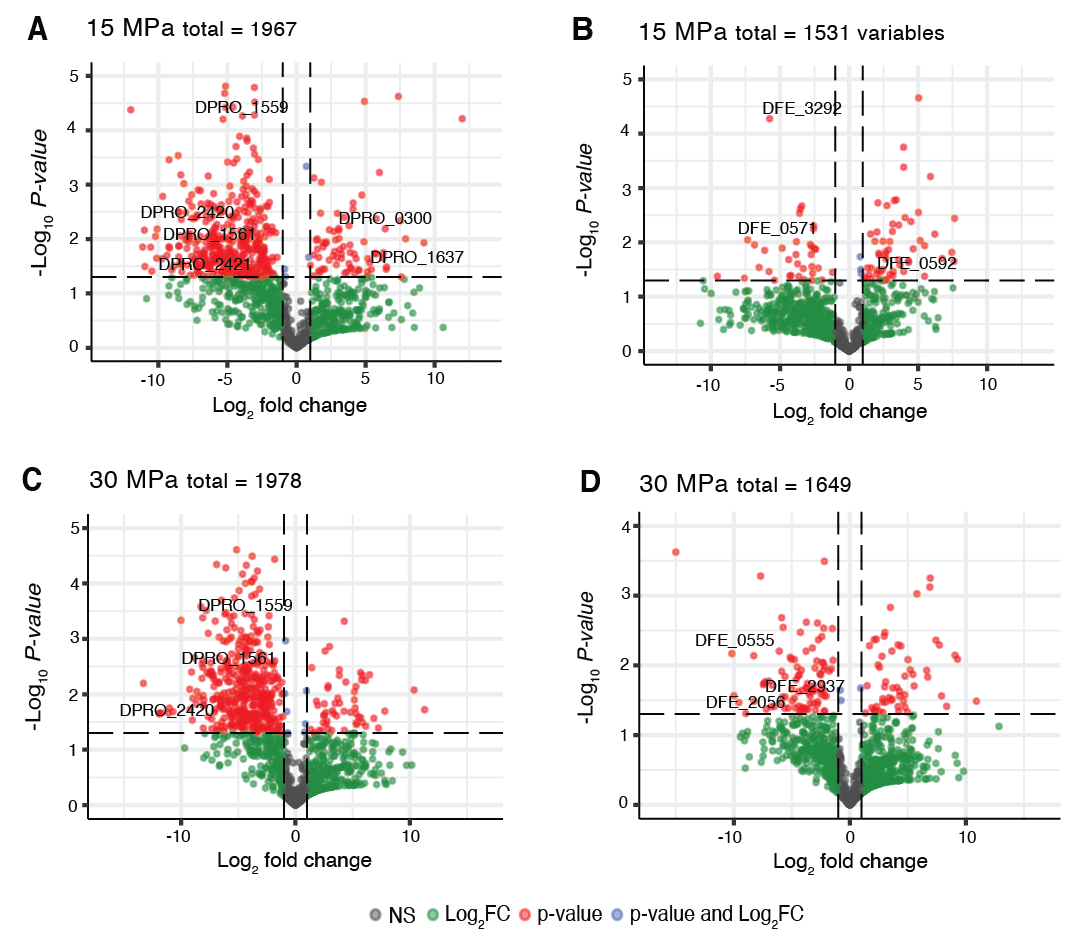


Figure S1. Volcano plots comparing differentially expressed proteins in biofilm over planktonic cells in *P. profundus* (A, C) *D. ferrophilus* (B, D) at 15 (A, B) and 30 (C, D) MPa respectively.

| COG categories | | *P. profundus* | | | | | | *D. ferrophilus* | | | | | |
| --- | --- | --- | --- | --- | --- | --- | --- | --- | --- | --- | --- | --- | --- |
|  |  | 0.1 MPa | 15 MPa | 30 MPa | 0.1 MPa | 15 MPa | 30 MPa | 0.1 MPa | 15 MPa | 30 MPa | 0.1 MPa | 15 MPa | 30 MPa |
| **J** | Translation, Ribosomal structure and biogenesis | 5 | 3 | 4 | 40 | 37 | 43 | 0 | 1 | 1 | 1 | 3 | 14 |
| **K** | Transcription | 8 | 5 | 3 | 34 | 37 | 33 | 5 | 3 | 6 | 3 | 3 | 4 |
| **L** | Replication, Recombination and Repair | 8 | 6 | 6 | 13 | 21 | 14 | 0 | 0 | 2 | 1 | 1 | 5 |
| **B** | Chromatin structure and dynamics | 0 | 0 | 0 | 0 | 0 | 0 | 1 | 1 | 0 | 0 | 0 | 0 |
| **D** | Cell cycle control, Cell division, Chromosonme partitioning | 2 | 1 | 1 | 8 | 7 | 6 | 0 | 0 | 1 | 0 | 0 | 1 |
| **V** | Defense mechanisms | 3 | 3 | 2 | 7 | 7 | 5 | 0 | 0 | 1 | 0 | 0 | 0 |
| **T** | Signal trasduction mechanisms | 15 | 11 | 11 | 53 | 48 | 56 | 6 | 11 | 6 | 8 | 4 | 14 |
| **M** | Cell wall/Menmbrane/Envelope biogienesis | 10 | 8 | 3 | 38 | 33 | 31 | 4 | 7 | 9 | 1 | 6 | 3 |
| **N** | Cell motility | 5 | 3 | 3 | 14 | 13 | 13 | 1 | 3 | 1 | 3 | 0 | 9 |
| **U** | Intracellular traffiking, Secretion, and Vesicular trasport | 2 | 1 | 1 | 5 | 4 | 6 | 0 | 1 | 0 | 1 | 1 | 2 |
| **O** | Post-translational modification, Protein turnover, Chaperones | 1 | 0 | 1 | 31 | 36 | 41 | 0 | 2 | 4 | 1 | 0 | 3 |
| **C** | Energy production and convertion | 1 | 0 | 1 | 80 | 71 | 68 | 6 | 8 | 2 | 7 | 12 | 26 |
| **G** | Carbohydrate transport and metabolism | 5 | 2 | 2 | 29 | 23 | 29 | 1 | 0 | 1 | 1 | 1 | 3 |
| **E** | Amino Acid transport and metabolism | 4 | 4 | 1 | 83 | 77 | 78 | 3 | 2 | 5 | 9 | 10 | 16 |
| **F** | Nucleotide transporta and metabolism | 0 | 0 | 0 | 28 | 26 | 30 | 3 | 1 | 2 | 4 | 2 | 9 |
| **H** | Coenzyme transport and metabolism | 9 | 6 | 5 | 38 | 39 | 35 | 0 | 1 | 1 | 0 | 2 | 8 |
| **I** | Lipid transport and metabolism | 1 | 1 | 1 | 13 | 12 | 8 | 0 | 1 | 1 | 0 | 0 | 2 |
| **P** | Inorganic ion transport and metabolism | 5 | 2 | 2 | 26 | 25 | 24 | 0 | 1 | 1 | 2 | 1 | 6 |
| **Q** | Secondary metabolites biosynthesis, transport and catabolism | 4 | 2 | 1 | 5 | 4 | 7 | 1 | 1 | 0 | 0 | 0 | 2 |
| **S** | Function unknown | 12 | 10 | 16 | 80 | 73 | 78 | 7 | 8 | 10 | 8 | 5 | 16 |

Table S1. Cluster of orthologous group (COG) categories and number of proteins differently expressed in biofilm against planktonic cells at 0.1, 15 and 30 MPa in *P. profundus* and *D. ferrophilus* cultures. In green proteins overexpressed in biofilm, in red proteins overexpressed by planktonic cells.

| ***P. profundus*** | | | ***D. ferrophilus*** | | |
| --- | --- | --- | --- | --- | --- |
| **Protein** | **log2FC** | **p_value** | **Protein** | **log2FC** | **p_value** |
| DPRO_2385 | -10.03 | 0.0309 | DFE_0173 | -1.41 | 0.4681 |
| DPRO_0300 | -6.72 | 0.2267 | DFE_0599 | 2.48 | 0.1391 |
| DPRO_1637 | 1.65 | 0.0527 | DFE_0653 | 3.30 | 0.0737 |
| DPRO_1513 | -4.99 | 0.0059 | DFE_1090 | 1.57 | 0.1113 |
| DPRO_1866 | -0.04 | 0.8499 | DFE_1385 | 1.36 | 0.0139 |
| DPRO_0986 | -5.72 | 0.0004 | DFE_1444 | 6.24 | 0.1307 |
| DPRO_3650 | -8.66 | 0.0504 | DFE_2667 | 5.34 | 0.0117 |
| DPRO_0194 | 5.59 | 0.0098 | DFE_2829 | 3.08 | 0.0378 |
| DPRO_2419 | 6.41 | 0.0026 | DFE_3137 | -0.23 | 0.8622 |
| DPRO_2421 | 3.91 | 0.0000 | DFE_3175 | -1.03 | 0.1650 |
| DPRO_1445 | 6.11 | 0.0000 |  |  |  |
| DPRO_1446 | 6.48 | 0.0004 |  |  |  |
| DPRO_1558 | 4.16 | 0.0027 |  |  |  |
| DPRO_1559 | 5.02 | 0.0000 |  |  |  |
| DPRO_1560 | 3.90 | 0.0046 |  |  |  |
| DPRO_1561 | 4.55 | 0.0007 |  |  |  |
| DPRO_1562 | 2.56 | 0.0001 |  |  |  |
| DPRO_1563 | 3.21 | 0.0004 |  |  |  |
| DPRO_1564 | 3.17 | 0.0001 |  |  |  |
| DPRO_1936 | 27.07 | 0.0138 |  |  |  |
| DPRO_1937 | 4.85 | 0.0005 |  |  |  |
| DPRO_1938 | 4.52 | 0.0023 |  |  |  |
| DPRO_1939 | 27.48 | 0.0076 |  |  |  |
| DPRO_2838 | -2.57 | 0.2291 |  |  |  |
| DPRO_2839 | 4.74 | 0.0017 |  |  |  |
| DPRO_2841 | 7.01 | 0.0021 |  |  |  |
| DPRO_2842 | 3.49 | 0.0078 |  |  |  |
| DPRO_2843 | 4.23 | 0.0650 |  |  |  |
| DPRO_2844 | 4.97 | 0.0231 |  |  |  |
| DPRO_2846 | 27.77 | 0.0031 |  |  |  |
| DPRO_2847 | 4.75 | 0.0039 |  |  |  |
| DPRO_2850 | 4.16 | 0.0005 |  |  |  |

Table S2. Differential expression and statistical significance of selected proteins in planktonic cells against biofilm at 0.1 MPa. Positive log2FC indicates higher expression in planktonic cells.

| **MHP induced** | **HHP induced** | **MHP inhibited** | **HHP inhibited** |
| --- | --- | --- | --- |
| DPRO_0125 | DPRO_0348 | DPRO_0660 | DPRO_0225 |
| DPRO_0288 | DPRO_0447 | DPRO_2593 | DPRO_0275 |
| DPRO_0967 | DPRO_0788 | DPRO_2810 | DPRO_0296 |
| DPRO_1061 | DPRO_0858 | DPRO_3101 | DPRO_0985 |
| DPRO_1608 | DPRO_2241 | DPRO_3322 | DPRO_1013 |
| DPRO_1934 | DPRO_2537 | DPRO_3642 | DPRO_1357 |
| DPRO_2179 | DPRO_2758 |  | DPRO_1490 |
| DPRO_2284 | DPRO_2771 |  | DPRO_1500 |
| DPRO_2527 | DPRO_2849 |  | DPRO_1743 |
| DPRO_2577 | DPRO_3106 |  | DPRO_1787 |
| DPRO_2213 | DPRO_3335 |  | DPRO_1908 |
| DPRO_2483 | DPRO_3551 |  | DPRO_2032 |
| DPRO_3179 | DPRO_3674 |  | DPRO_2830 |
|  | DPRO_3758 |  | DPRO_3437 |
|  | DPRO_3799 |  |  |
|  | DPRO_3851 |  |  |

Table S3. List of the significantly differently expressed proteins in *P. profundus* biofilms at 15 MPa (MHP) and 30 MPa (HHP).

| **MHP induced** | **HHP induced** | **MHP inhibited** | **HHP inhibited** |
| --- | --- | --- | --- |
| DFE_0052 | DFE_0157 | DFE_0115 | DFE_0207 |
| DFE_0187 | DFE_0299 | DFE_0001 | DFE_1109 |
| DFE_0667 | DFE_0624 | DFE_0318 | DFE_1546 |
| DFE_0915 | DFE_0627 | DFE_0333 | DFE_2114 |
| DFE_1038 | DFE_0599 | DFE_0417 | DFE_2227 |
| DFE_1090 | DFE_0704 | DFE_0439 | DFE_2389 |
| DFE_1818 | DFE_0767 | DFE_0551 | DFE_2702 |
| DFE_1836 | DFE_1257 | DFE_0791 | DFE_3007 |
| DFE_2001 | DFE_1541 | DFE_1037 | DFE_3338 |
| DFE_2538 | DFE_1627 | DFE_1058 |  |
| DFE_2682 | DFE_1985 | DFE_1152 |  |
| DFE_2765 | DFE_2351 | DFE_1237 |  |
| DFE_2858 | DFE_2359 | DFE_1239 |  |
| DFE_2837 | DFE_2340 | DFE_1382 |  |
| DFE_2840 | DFE_2761 | DFE_1602 |  |
|  | DFE_2926 | DFE_1910 |  |
|  |  | DFE_2006 |  |
|  |  | DFE_2044 |  |
|  |  | DFE_2062 |  |
|  |  | DFE_2087 |  |
|  |  | DFE_2367 |  |
|  |  | DFE_2667 |  |
|  |  | DFE_2829 |  |
|  |  | DFE_3005 |  |
|  |  | DFE_3301 |  |

Table S4. List of the significantly differently expressed proteins in *D. ferrophilus* biofilms at 15 MPa (MHP) and 30 MPa (HHP).
